# MATES in Construction: Impact of a Multimodal, Community-Based Program for Suicide Prevention in the Construction Industry

**DOI:** 10.3390/ijerph8114180

**Published:** 2011-11-07

**Authors:** Jorgen Gullestrup, Belinda Lequertier, Graham Martin

**Affiliations:** 1 MATES in Construction, Level 1, 35 Astor Terrace, Spring Hill, Queensland 4004, Australia; E-Mail: jorgen@matesinconstruction.org.au; 2 Centre for Psychiatry and Clinical Neuroscience, The University of Queensland, K Floor, Mental Health Centre, Royal Brisbane and Women’s Hospital, Herston, Queensland 4006, Australia; E-Mail: b.lequertier@uq.edu.au

**Keywords:** suicide, prevention, men, male health, gender, construction industry

## Abstract

A large-scale workplace-based suicide prevention and early intervention program was delivered to over 9,000 construction workers on building sites across Queensland. Intervention components included universal General Awareness Training (GAT; general mental health with a focus on suicide prevention); gatekeeper training provided to construction worker volunteer ‘Connectors’; Suicide First Aid (ASIST) training offered to key workers; outreach support provided by trained and supervised MIC staff; state-wide suicide prevention hotline; case management service; and postvention support provided in the event of a suicide. Findings from over 7,000 workers (April 2008 to November 2010) are reported, indicating strong construction industry support, with 67% building sites and employers approached agreeing to participate in MIC. GAT participants demonstrated significantly increased suicide prevention awareness compared with a comparison group. Connector training participants rated MIC as helpful and effective, felt prepared to intervene with a suicidal person, and knew where to seek help for a suicidal individual following the training. Workers engaged positively with the after-hours crisis support phone line and case management. MIC provided postvention support to 10 non-MIC sites and sites engaged with MIC, but not yet MIC-compliant. Current findings support the potential effectiveness and social validity of MIC for preventing suicide in construction workers.

## 1. Introduction

Suicide represents a substantial health and social issue for Australia, with the Department of Health and Ageing estimating that suicide comprises 3.9% of the total burden of disease in terms of years of life lost [1]. Australian Bureau of Statistics (ABS, 2011) figures for 2009 indicate that 2,130 Australians completed suicide (9.6 per 100,000), accounting for 1.5% of total deaths recorded, 24.0% of all deaths due to injuries, and exceeding the 1,417 deaths due to transport accidents [2].

The impact of gender on suicide rates has previously been acknowledged, both internationally [3] and in Australia, where 76.6% of 2009 suicides were male [2]. Drawing on data from the National Mortality Database, the Australian Institute of Health and Welfare reported that suicide was the leading cause of death for males aged 25–44 years in 2007. Suicide ranks second to coronary heart disease in its contribution to potential years of life lost (PYLL) by Australian males [4].

An emerging area of interest in suicide research is the impact of employment status and industry on rates of suicide. While being employed is associated with reduced risk of suicide overall [5,6], recent evidence suggests suicide rates are differentially distributed across industry and occupational groups. Australian males in manual occupations were more likely to have completed suicide between 1966 and 2001 than their non-manual worker counterparts [7]. This gap may be widening, with rates of suicide for manual workers increasing over the period examined, while rates for non-manual workers remained stable.

Research in Australia and abroad has demonstrated elevated rates of suicide amongst tradesmen and construction workers compared with the general working male population [8–11]. To investigate industry-specific patterns in Queensland suicides, Anderson and colleagues [5] compared data from 7,652 suicides in the Queensland Suicide Register with population data from the ABS for 1990–2006. For individuals 15–64 years, the greatest risk of suicide was in Queensland agricultural, transport and construction industries, with all suicides by construction workers being male.

Following a Royal Commission into the Building and Construction industry report identifying that 41% of all death claims made on behalf of Queensland construction workers over a four-month period were attributed to suicide [12], the Queensland Commercial Building and Construction Industry commissioned the Australian Institute for Suicide Prevention and Research (AISRAP) to investigate incidence and correlates of suicide within the industry. The study estimated suicide rates for construction workers at 40.3 per 100,000, significantly above the overall national rate for males (16.8 per 100,000) [13,14]. In particular, the rate for 15–24 year olds (58.6 per 100,000 people) represented a three-fold increased risk of suicide compared with male Australians of that age group generally, and in Queensland. Compared with other industries, construction workers were significantly more likely to have consumed alcohol immediately prior to death, and experienced relationship problems and multiple stressful life events in the months prior to death [13,14]. Construction workers aged 15–24 years who completed suicide were significantly more likely than non-construction counterparts to show evidence of untreated psychiatric conditions preceding death [13,14].

Focus group discussions with Queensland construction industry workers and representatives identified both industry-specific and more general factors that may contribute to increased risk, including work conditions, interpersonal relationship difficulties, marital breakdown and associated issues maintaining relationships with children, along with job insecurity and associated pressures [13,14]. Some studies suggest suicide rates for construction workers reduce markedly once demographic and socioeconomic variables are taken into account (gender, age and relationship status) [9,10,15,16], while others find the elevations robust despite controlling for these factors [6,8].

‘MATES in Construction’ (MIC) is a charity established in February 2008 by the Building Employees Redundancy Trust (BERT) to prevent suicide through implementing recommendations of the AISRAP study [14]. MIC is funded from several sources; 40% funding from BERT, 25% from the Queensland Government, and 35% from a variety of industry funds including unions, employer associations and contractors. MIC is a multimodal prevention and early intervention program, consistent with the Living Is For Everyone (‘LIFE’) strategy [17] and Mrazek and Haggerty’s [18] spectrum of prevention and intervention, and includes the following objectives:

Universal Prevention:

- promote awareness amongst construction workers about mental illness and suicide in the construction industry, warning signs for suicide and the preventability of suicide
- reduce stigma associated with mental illness, suicide and help-seeking

Selective Prevention:

- enhance symptom identification by implementing a volunteer gatekeeper program within the construction industry community
- improve access and engagement with specialised services and programs for specific difficulties (e.g., drug and alcohol problems, separated fathers without custody of their children)

Indicated Prevention:

- improve access to mental health intervention through flexible delivery of outreach and case management support for those at risk
- ensure support provided is mindful of the context and needs of the construction industry population, to maximise engagement
- encourage workers to access 24-hour telephone crisis support

Treatment:

- facilitate referral for at-risk workers to appropriate local services
- collaborate with services utilised by at-risk workers

Postvention:

- ensure ongoing support for workers who have attempted suicide
- offer bereavement support following suicide of an industry worker

We present preliminary evaluation of the effectiveness of MIC for the period from April 2008 to November 2010. While the primary goal of suicide prevention programs is to reduce suicidal behaviour, the low base rate of suicide and complexities associated with gathering accurate data regarding attempted suicide suggest that these outcomes are less meaningful for evaluation of a program in the short term [19]. In line with AISRAP recommendations [14], we examined the impact of MIC on short- and medium-term indicators of effectiveness, including knowledge of suicide prevention and support services, and help-seeking behaviour. Additionally, it has been recommended that consideration of effectiveness of an intervention should include examination of its social validity, that is, the degree to which the focus and procedures involved are viewed by the target population as acceptable and of social importance [20,21]. Given evidence that male construction workers who suicide are less likely to have sought or accessed assistance for mental health difficulties, we argue that social validity is crucial when evaluating MIC.

## 2. Methods

### 2.1. Design

Public and private sector construction sites across Queensland were recruited to participate in MATES in Construction. Gender was not recorded for MIC participants; however, Queensland Census 2006 findings indicate that 85.6% of Queensland construction industry are male [22]. Given low literacy rates in the target population, participants in MIC training were informed verbally that completion of questionnaires was for the purpose of program evaluation; anonymous completion of questionnaires was taken as informed consent. Due to the itinerant nature of the industry, some participants may have completed GAT more than once; every effort was made to remove duplicates.

A non-equivalent group comparison design was used to evaluate effectiveness of General Awareness Training (GAT) on participants’ mental health and suicide prevention knowledge. Our main test group data was collected from March 2009 to November 2010 and comprises 7311 participants who completed questions 1–5 inclusive of the GAT questionnaire (‘pre-GAT’), and then questions 6–10 inclusive after they had received the information from GAT (‘post-GAT’). GAT questionnaire data collection commenced following an initial GAT pilot phase involving 1264 workers.

Comparison data was collected from two sites in May 2010 (n = 355), with participants completing all 10 GAT questions prior to exposure to the GAT course, hence responding to questions 6–10 without intervening information. In the absence of demographic data being available for the GAT and comparison groups, we have assumed these two site groups are equivalent in terms of age range, marital status, and income to our test group, given they were drawn from the same target population of Queensland construction workers randomly selected on the basis of work site.

### 2.2. Intervention

MIC can be considered an industry-based, but workplace-focused program, with components delivered at construction sites or company offices, except Suicide First Aid training which was delivered in a training facility. A significant commitment from building site management to the program was crucial, both to communicate organisational investment in addressing the issue of suicide, and also considering that training was completed during work hours. We acknowledge this commitment occurred in the context of economic and industry pressures.

#### 2.2.1. General Awareness Training

GAT [23] is a 45-minute training session provided to all construction workers on sites recruited to the study, provided by any of six MIC staff, including field officers and management. It is provided as a stand-alone session, or as a component of the Life Skills Toolbox, a training program for apprentices. This universal intervention increases awareness of mental health and suicide in the industry, considers them a workplace health and safety issue, improves knowledge regarding warning signs, and encourages workers to offer support to co-workers who display warning signs of possible increased suicide risk. Workers completing GAT are provided with a white MIC sticker to wear on their hard hat. To be ‘MIC-compliant’ requires that all workers on a given worksite are exposed to GAT, with an 80% training level maintained despite staff turnover.

#### 2.2.2. Connector Training

Connectors are recruited through a question on the GAT feedback form inviting expressions of interest in volunteering for this role. A Connector (visually identifiable by a green MIC sticker on their hard hat) is defined as “a mate who can keep you safe while connecting you to help”. They receive four hours of training [24], incorporating Livingworks safeTALK [25], to gain additional knowledge and skills at identifying warning signs of suicidality, as well as strategies for engaging a co-worker and establishing if risk may be present. At the conclusion of training, participants complete a role-play exercise allowing them to rehearse strategies learned. The Connector role is to assist an at-risk worker to access help via an ASIST-trained worker (see below), MIC Field Officer or Case Manager. The MIC objective is to have 1 in 20 workers on every site trained as Connectors, with these ideally representing a diverse range of roles, ages and levels of experience to increase the likelihood an at-risk worker will be noticed and supported by a Connector, and will find a Connector with whom they feel comfortable. Connectors are supported by Field Offers through opportunities to debrief following an intervention and through regular Connector meetings.

#### 2.2.3. Suicide First Aid

Participants at GAT and Connector training are informed about optional Suicide First Aid training. Once a Connector identifies an at-risk individual, he supports him until an individual trained in Suicide First Aid is available to offer intervention. All sites are encouraged to support interested workers to receive training in Suicide First Aid, through the Livingworks’ 2-day ‘Applied Suicide Intervention Skills Training’ (ASIST) [26,27]. This component is mandatory for rural and remote sites where ASIST-trained MIC staff and other agencies may be less available. ASIST is funded by industry unions or employers, and is considered an important component for long-term sustainability of MIC. A recent review found support for effectiveness and acceptability of this training [26].

#### 2.2.4. Field Officers

During the course of the study, 3.5 Field Officers were employed in Queensland–to increase awareness of the program, recruit new construction sites, and provide ongoing support to MIC sites through fortnightly site visits, establishing and maintaining relationships with workers on-site, and debriefing Connectors. They also have a role in reducing stigma associated with help-seeking. Field Officers are ASIST-trained and may intervene to provide direct support to suicidal workers.

#### 2.2.5. Case Management

The role of the MIC Case Manager is considered key to enhancing engagement of workers with external services, providing a ‘safety net’ between agencies to ensure continuity of care and follow-up, to ensure that workers’ needs are being met, advocating for further assistance where necessary. MIC employs 1.5 case managers.

#### 2.2.6. Suicide Prevention Hotline

A 24-hour emergency help-line, staffed by trained mental health professionals, was established to provide assistance to suicidal individuals, or those concerned about the risk of another person.

#### 2.2.7. Specialised Intervention

MIC Field Officers and Case Managers assess and refer at-risk workers to appropriate external support services, mental health or counselling services, financial counselling, and drug and alcohol intervention; this includes the existing Employee Assistance Program (Converge International) contracted by BERT. MIC also facilitates the ‘Staying Connected’ program for single fathers, for which participants are invited to register interest on their GAT questionnaire.

#### 2.2.8. Postvention

MIC offers discussion with building site management regarding support for workers after the suicide of a colleague, as well as providing written and verbal information about common grief reactions. MIC can facilitate discussions amongst workers closest to the deceased person and organises counsellor support for the site. The Field Officer continues to provide support for the site through visits and phone calls up to several months following an incident, depending on circumstances.

### 2.3. Measures

#### 2.3.1. Social Validity and Acceptability of MATES in Construction

The rate of participation in MIC by construction sites was examined as an indicator of social validity and acceptability of MIC to the QLD construction industry. Additionally, rate of worker participation in the General Awareness and Connector Training was considered an indicator of acceptability of the intervention to the industry.

#### 2.3.2. General Awareness Training Questionnaire

Effectiveness of GAT was assessed using a questionnaire developed to measure suicide and suicide prevention awareness and beliefs, drawing on the ‘Miss/Dismiss/Avoid’ component of safeTALK [25] training, and covering common suicide myths challenged during GAT. Participants indicated agreement to 10 statements on a 5-point Likert scale, from *strongly agree* to *strongly disagree* (see Table 1). Cronbach’s alpha was used to provide a measure of internal consistency for pre- and post- questionnaires. Given disparate numbers between test and comparison samples, we chose to use the Mann-Whitney U statistical test to determine difference; all tests were two-tailed and corrected for ties.

#### 2.3.3. Connector Training Feedback Forms

On completion of Connector training, participants completed a series of evaluation questions for the safeTALK and Connector components of the training (refer Table 3). These items were rated on a 4-point, forced-choice Likert Scale from *strongly agree* to *disagree*. Diffusion intention [28] was measured by a *yes* or *no* response to “I intend to tell others that they will benefit from this training”. Participants gave ratings on a 4-point Likert scale from *well prepared* to *not prepared* for the statement “How prepared do you now feel to talk directly and openly to a person about their thoughts of suicide?” The helpfulness of the training was rated by participants on a scale of 1 (*not at all helpful*) to 5 (*very helpful*). Qualitative feedback was also invited on the feedback forms, but was not examined for the current study.

## 3. Results

### 3.1. Social Validity and Acceptability of MATES in Construction

**Figure 1:**
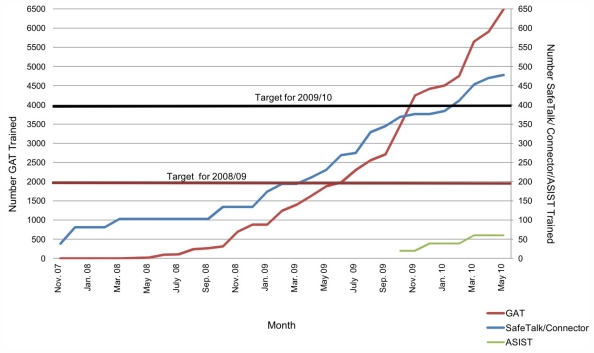
Progress towards MIC training targets.

During the period of the study, 54 of 83 building sites approached by MIC (64%) agreed to participate in the program. As at 30th November 2010, four sites were considered ‘MIC compliant’, indicating that at least 80% of workers completed GAT, together with one trained Connector per 20 workers on site. Twenty-nine sites had completed GAT and were progressing towards MIC compliance, and 21 prospective sites had indicated interest in MIC. Figures on MIC compliance are an underestimate given that worksites are time-limited, and sites may have achieved compliance, but were then closed. Participation in most aspects of MIC has grown exponentially since the beginning of the program, as depicted in Figure 1.

### 3.2. Effectiveness of General Awareness Training

On the pre-GAT measure of suicide and prevention awareness (Cronbach α = 0.75), the majority of GAT participants *disagreed* or *strongly disagreed* with each of the statements (see Table 1): “you can’t stop people who really want to suicide” (52.7%), “suicide is a private issue that should be dealt with in the home only” (81.5%), “suicide always occurs without warning signs” (62.4%), “talking about suicide can cause suicide” (59.8%), and “women are more at risk of suicide than men” (71.2%). An initial test examined baseline suicide prevention awareness, comparing test subjects (7311) with comparison subjects (355) using Mann-Whitney *U* test. Comparing responses to each question in turn, no significant differences were found between test and comparison groups on the first five (pre-GAT) questions.

Responses by GAT participants on the post-GAT questionnaire (Cronbach α = 0.88) indicated that most participants *agreed* or *strongly agreed* with “people considering suicide often send out warning signs or invitations” (73.4%), “suicide is everyone’s business” (80.3%), “most people who suicide really don’t want to die” (76.5%), “poor mental health is a workplace health and safety issue” (80.9%), and “the construction industry must do something to reduce suicide rates” (87.4%).

Mann-Whitney *U* tests found significant intervention effects with stronger agreement from GAT participants on all five post-GAT questions than comparison group participants. The results of these analyses were: Question 6 (“people considering suicide often send out warning signs or invitations”), *U =* 1090887.50, *z =* −4.382, *p =* 0.00, *r* = 0.05; Question 7 (“suicide is everyone’s business”), *U =* 1033887.00, *z =* −6.05, *p =* 0.00, *r* = 0.07; Question 8 (“most people who suicide really don’t want to die”), *U =* 924475.00, *z =* −8.83, *p =* 0.00, *r* = 0.10; Question 9 (“poor mental health is a workplace health and safety issue”), *U =* 1149071.50, *z =* −2.68, *p =* 0.00, *r* = 0.03; and Question 10 (“the construction industry must do something to reduce suicide rates”) *U =* 1007089.00, *z =* −6.76, *p =* 0.00, *r* = 0.08.

Due to disparity of sample size between the GAT and comparison groups, the Mann-Whitney test was applied to a subsample of 355 GAT participants who completed GAT during the same period that the 355 comparison participants’ data were collected. The purpose of this was to reduce any influence of contextual factors that could threaten the external validity of findings, for example the weather or working conditions during that month, or diffusion of suicide prevention awareness through the industry since the commencement of the MIC program, with possible repeat exposure to GAT due to the itinerant nature of the population, and other contextual, societal or industry factors. In the absence of data to confirm this, it was assumed that diffusion of awareness and repeat exposure would occur randomly, with these issues affecting GAT and comparison groups equally.

Analysis of pre-GAT results from this subsample replicated the findings based on the full GAT sample, namely, an absence of significant differences between groups on the baseline questions. The post-GAT findings also replicated the large sample analyses, with GAT participants demonstrating significantly stronger agreement with items assessing suicide awareness than comparison participants on Question 6 (*U =* 52202.00, *z =* −3.76, *p =* 0.00, *r* = 0.13, *N=*701), Question 7 (*U =* 45199.00, *z =* −6.54, *p =* 0.00, *r* = 0.25, *N =* 701), Question 8 (*U =* 43568.50, *z =* −7.12, *p =* 0.00, *r* = 0.27, *N =* 700), Question 9 (*U =* 51516.50, *z =* −3.93, *p =* 0.00, *r* = 0.15, *N =* 699), Question 10 (*U =* 44496.00, *z =* −6.86, *p =* 0.00, *r* = 0.26, *N =* 699). Data from these analyses are presented in Table 2.

### 3.3. Effectiveness of Connector Training

Data were available for 696 participants (96%) who completed Connector Training between October 2008 and November 2010. Although data was not collected on referral pathways to Connector Training, many of these were likely to have been recruited through the GAT course, with 7% (n = 602) of all GAT participants indicating on their GAT forms a willingness to complete the Connector training and to perform this role on site. Data was available for a total of 424 safeTALK feedback forms and 604 Connector feedback forms. Responses to items on the Connector forms are presented in Table 3, demonstrating strong endorsement of items reflecting the social importance and perceived effectiveness of the MIC program.

**Figure 2:**
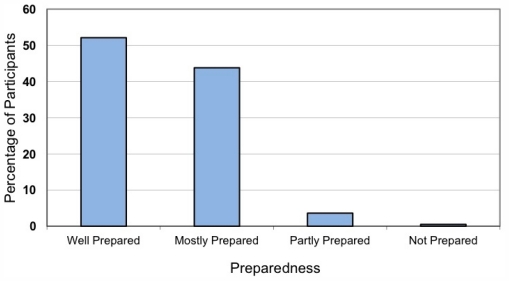
Connector preparedness to speak to someone about suicide.

Following Connector training, 98.8% of participants *agreed* or *strongly agreed* that they “know where and how to get help now” (refer Table 3). On the evaluation form for the safeTALK component of Connector Training (n = 424), 96% of participants reported feeling *well prepared* or *mostly prepared* to “talk directly and openly to a person about their thoughts of suicide” (Figure 2). With regard to helpfulness of the training, 74.1% of respondents rated training as 5 (*very helpful)* on a scale from 1 (*not at all helpful*) to 5 (*very helpful*). Ratings indicated that 98.6% (n = 418) of participants intended to tell someone about the program.

### 3.4. Help-Seeking Behaviour and Engagement with Intervention

**Figure 3:**
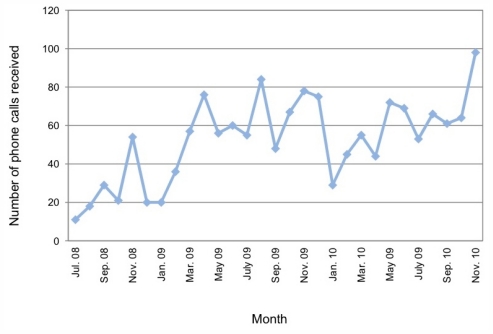
Calls to after-hours crisis support line.

The MIC emergency help-line received 1521 after-hours calls between July 2008 and November 2010, with patterns of usage over time indicated in Figure 3. We note this excludes calls responded to by MIC staff during business hours, which is not available. Additionally, 328 (4%) GAT participants requested a follow-up phone call from a Field Officer by indicating this on the evaluation form following the training.

A total of 674 workers accessed case management from MATES in Construction between April 2008 and November 2010, which represents 7.2% of MIC participants. Of these, 44% were self-referred (n = 287), with referrals also made by unions (n = 94), Connectors (n = 74), employers (n = 67), other service providers (n = 57), family (n = 23), training organisations (n = 15) or due to a critical incident response (n = 7). Thirty of these clients were referred to case management through the apprenticeship-based resilience program, Life Skills Toolbox. A majority of clients were based in Brisbane (n = 413), whilst case management was also provided to clients living in the Gold Coast (n = 139), Sunshine Coast (n = 8), Central Queensland (n = 7), regional Queensland (n = 66) and interstate (n = 10).

**Figure 4:**
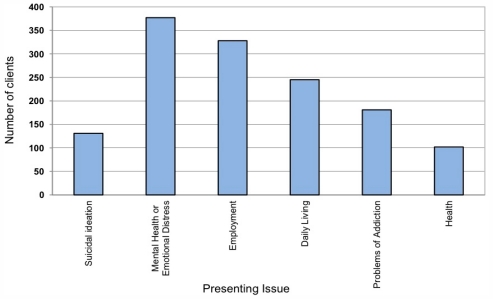
Presenting issues on referral to case management.

As depicted in Figure 4, case management clients sought assistance from MIC for a variety of issues, with many presenting multiple areas of concern. Active suicidal ideation was reported by 101 referred clients, with 30 clients requiring suicide intervention. Case management clients were eventually referred to any of a variety of specialist services, depending on the nature of the presenting difficulties. Forty-one percent of case management clients were referred to Converge International (n = 274), an employee-assistance counselling organisation contracted to provide services to MIC clients. Liaison or referral also occurred with unions, financial services, various Government Departments, medical services, and legal professionals.

### 3.5. Postvention

Postvention support was provided to 10 building sites in Queensland. Eight of these involved support following a suicide, one followed a lethal workplace accident, and one following an accidental death outside work hours. Postvention following the accidental death occurred on a site that was MIC-compliant at the time, but is now closed. Four postventions occurred on sites where some workers had received GAT, and the remaining five occurred with sites not participating in MIC. None of the deceased workers had attended GAT, and it is noted that suicide postvention support was the impetus for two sites to eventually engage with MIC.

## 4. Discussion

The current study found evidence to support the social validity and effectiveness of the MATES In Construction program for the Queensland Construction Industry. A positive response from construction industry employers has been critical to program implementation, with this requiring staff to “down tools” in order to participate in MIC components, but also to convey organisational support for suicide prevention and MIC. This strong commitment to the program by the industry was assisted by MIC being partially funded and indirectly overseen by both Unions and Employer Associations through BERT, as well as frequent consultation between MIC and industry representatives throughout program development and implementation. Following GAT, a majority of the 7311 participants indicated high levels of agreement that mental health and suicide prevention is a workplace health and safety issue, and should be addressed by the construction industry. The number of volunteers to become Connectors exceeded MIC compliance standards of one Connector for every 20 construction workers. A majority of Connector Training participants reported that they could see how MIC would be effective in saving lives on site and that they appreciated the importance of meeting MIC compliance objectives. Considering that at-risk male construction workers may be less likely to seek assistance for mental health difficulties [13], the level of engagement by participants of MIC, both in terms of sheer numbers and positive ratings on questionnaires, suggests that MIC is a feasible and acceptable intervention for reducing the suicide rate within this difficult-to-engage population.

In addition to its social validity, analysis of short- and medium-term indicators provides evidence of MIC effectiveness, as per AISRAP [14] recommendations. GAT participants demonstrated significantly increased ratings of suicide prevention awareness compared to the non-GAT comparison group. Given effect sizes were small, the main impact of GAT training may be through the social legitimisation of building industry concern about suicide prevention. Connector training participants’ ratings of the training as helpful, together with their positive evaluations of their help-seeking knowledge and preparedness to intervene with a suicidal individual following exposure to the training, also provides qualitative evidence of the potential effectiveness of this component. Furthermore, a majority of participants reported that they intended to tell others about the training, which may indicate potential diffusion effects of suicide prevention awareness throughout the wider community [28].

These findings indicated positive engagement by MIC participants with support options presented by the program. An increasing number of participants accessed the MIC emergency help-line and 4% of GAT participants (n = 328) requested a follow-up call from a MIC staff member following involvement in this training. Case management support was accessed by 7.2% of workers on involved sites. This compares favourably with the rate of approximately 5% of Australian men with a mental health condition in the 12 months prior to 2007 who accessed professional support according to the Australian Bureau of Statistics [29]. Many case management referrals were attributed to MIC initiatives, including referrals from Connectors, the Lifeskills Toolbox program for apprentices, or by indicating on GAT feedback forms. At 44%, self-referrals represented the greatest proportion of referrals to case management, which reflects positively on the mental health awareness and help-seeking behaviour by construction workers participating in MIC compared with the historically low engagement with mental health services for this population compared with non-construction worker peers [13,14].

Despite MIC being a suicide prevention program, the majority of referrals to case management were for issues other than suicidality. This is consistent with research suggesting that individuals are more likely to seek professional assistance for an emotional or personal problem than for suicidal ideation [30,31]. Accordingly, it is possible that MIC may have an impact on suicide rates longitudinally through several mechanisms: by improving awareness and reducing stigma associated with both mental health and help-seeking, whilst also facilitating access to services for early intervention targeting salient risk factors for this population, such as substance abuse and family issues [13].

The current findings should be considered in the context of methodological limitations. As the GAT questionnaire is contained in the workbook accompanying training, suicide prevention awareness ratings may have been influenced by workbook content. While the impact of this on the validity of GAT effectiveness findings is reduced due to comparison participants also completing questions within the workbook, descriptive data concerning baseline attitudes and knowledge may overestimate actual community awareness, possibly through a ceiling effect. Additionally, due to the itinerant nature of the industry, some GAT participants may have already completed the program, despite efforts to minimise duplication in the database. While it is likely that repeat exposures to GAT would be randomly distributed throughout the GAT and comparison samples, any impact on findings due to non-random distribution cannot be ruled out. In terms of influence of MIC on help-seeking, data on baseline levels of engagement with professional mental health and other support services would enable impact on help-seeking behaviours to be examined. For example, it may be useful to investigate whether those who engage with case management would have accessed support elsewhere, or at all, had they not been involved in MIC. Finally, the reliability and validity of our findings can be improved in future research by using standardised questionnaires, for example, to assess suicide awareness and help-seeking intentions or behaviour [32–34].

Comparison of GAT post-intervention ratings with a non-GAT comparison sample, to control for contextual confounding, demonstrated an intervention effect; however, in the absence of demographic data, it is impossible to conclude the equivalence of the two groups, hence findings should be interpreted with caution. It will be important to support the current preliminary findings with further evaluation employing a cluster-randomised repeated measures design. It is recommended that longitudinal follow-up examine the durability of intervention effects, to determine whether booster sessions of GAT or Connector Training are required. Investigation of the relative impact of the different program components may assist to maximise effectiveness and efficiency of MIC. Similarly, it may be helpful for future evaluations to consider how MIC may best be structured and delivered in future, as an increasing proportion of workers become GAT and Connector trained. Finally, while established risk and protective factors associated with suicide are often the best available outcome variables when evaluating suicide prevention programs in the short- and medium-term, the impact of MIC on actual rates of suicide would be important to examine once sufficient participant numbers enable this to be statistically meaningful.

## 5. Conclusions

The elevated rate of suicide amongst Queensland construction industry workers has captured the attention of researchers and industry leaders alike. MATES in Construction was developed using evidence-based suicide prevention principles consistent with the Living Is For Everyone (‘LIFE’) strategy [17] and Mrazek and Haggerty’s [18] spectrum of prevention and intervention. There is evidence to support the social validity and effectiveness of MIC for improving suicide and mental health awareness, help-seeking behaviour, and treatment engagement, thereby reducing the suicide risk for construction workers in Queensland. Further research using a longitudinal, cluster-randomised repeated-measures design is recommended to further support for the effectiveness of the MIC program.

## Figures and Tables

**Table 1:** General awareness training participant responses pre-training.

Question	Response Option					Missing values

	Strongly Agree	Agree	Maybe	Disagree	Strongly Disagree	
**1.** You can’t stop people who really want to suicide	264 (3.7%)	1161 (16.0%)	1965 (27.4%)	2551 (35.6%)	1233 (17.2%)	137 (1.9%)
**2.** Suicide is a private issue that should be dealt with in the home only	259 (3.6%)	413 (5.8%)	650 (9.1%)	3331 (46.5%)	2509 (35.0%)	149 (2.0%)
**3.** Suicide always occurs without warning signs	192 (2.7%)	817 (11.4%)	1685 (23.5%)	3264 (44.1%)	1366 (18.5%)	157 (2.1%)
**4.** Talking about suicide can cause suicide	147 (2.1%)	607 (8.5%)	2119 (29.6%)	2920 (40.8%)	1356 (19.0%)	165 (2.2%)
**5.** Women are more at risk of suicide than men	189 (2.6%)	365 (5.1%)	1505 (21.0%)	3410 (47.7%)	1685 (23.6%)	157 (2.1%)
**6.** People considering suicide often send out warning signs or invitations	1669 (23.3%)	3594 (50.1%)	1077 (15.0%)	630 (8.8%)	202 (2.8%)	139 (1.9%)
**7.** Suicide is everyone’s business	2491 (34.7%)	3279 (45.6%)	637 (8.9%)	554 (7.7%)	224 (3.1%)	126 (1.7%)
**8.** Most people who suicide really don’t want to die	1880 (26.2%)	3603 (50.3%)	978 (13.6%)	514 (7.2%)	193 (2.7%)	143 (2.0%)
**9.** Poor mental health is a workplace health and safety issue	2633 (36.7%)	3165 (44.1%)	583 (8.1%)	474 (6.6%)	315 (4.4%)	141 (1.9%)
**10.** The construction industry must do something to reduce suicide rates	3418 (47.8%)	2832 (39.6%)	310 (4.3%)	245 (3.4%)	342 (4.8%)	164 (2.2%)

**Table 2:** GAT and comparison group responses.

Question	GAT Group			Comparison Group			

	N	Mean	Median	N	Mean	Median	Mann-Whitney *p*-value
1. You can’t stop people who really want to suicide	7174	3.46	4.00	350	3.37	3.00	0.08
2. Suicide is a private issue that should be dealt with in the home only	7162	4.04	4.00	351	4.17	4.00	0.13
3. Suicide always occurs without warning signs	7158	3.64	4.00	352	3.75	4.00	0.06
4. Talking about suicide can cause suicide	7149	3.66	4.00	351	4.17	4.00	0.85
5. Women are more at risk of suicide than men	7154	3.84	4.00	352	3.93	4.00	0.16
6. People considering suicide often send out warning signs or invitations a	7172	3.82	4.00	349	3.66	4.00	0.00
7. Suicide is everyone’s business a	7185	4.01	4.00	350	3.71	4.00	0.00
8. Most people who suicide really don’t want to die a	7168	3.90	4.00	348	3.49	4.00	0.01
9. Poor mental health is a workplace health and safety issue a	7170	4.02	4.00	348	3.95	4.00	0.00
10. The construction industry must do something to reduce suicide rates a	7147	4.22	4.00	350	4.05	4.00	0.00

a Recoded scores are presented for items 6 to 10.

**Table 3:** Connector training feedback.

Question	Response Options			

	Strongly Agree	Agree	Partly Agree	Disagree
I can now see how MIC and the Connector training can save a life in my workplace	457 (75.3%)	142 (23.4%)	4 (0.7%)	0 (0.0%)
It is important to maintain 80% above Awareness on my site	450 (74.1%)	151 (24.9%)	2 (0.3%)	1 (0.2%)
MATES in Construction will work and could save lives on my site	440 (72.5%)	155 (25.5%)	2 (0.3%)	1 (0.2%)
I know where and how to get help now	453 (74.6%)	147 (24.2%)	2 (0.3%)	0 (0.0%)
